# Effective closure of post-gastric endoscopic submucosal dissection defect using anchor-pronged clip, string-clip, and detachable snare

**DOI:** 10.1055/a-2633-9122

**Published:** 2025-07-23

**Authors:** Shoichi Tanaka, Nanami Yamasaki, Kentaro Hamada, Kenji Ota, Yoshiaki Matsumura, Yukiteru Yanabe, Tsuyoshi Fujimoto

**Affiliations:** 1 Gastroenterology, National Hospital Organization Iwakuni Clinical Center, Iwakuni, Japan

Defect closure after gastric endoscopic submucosal dissection (ESD) is challenging owing to the thick wall, lumen width, and fragile mucosa. Although various clip techniques 1 2 3 4 have been reported, long-term effects of these methods on the maintenance of stomach closure remain unclear. We devised a robust defect closure method using an anchor-pronged clip (MANTIS clip; Boston Scientific, Waltham, Massachusetts, United States), string-clip, and detachable snare, which may facilitate the maintenance of long-term closure ( Video 1 ).

Defect closure after gastric ESD using MANTIS clip, string-clip, and detachable snare.Video 1

**Fig. 1:**
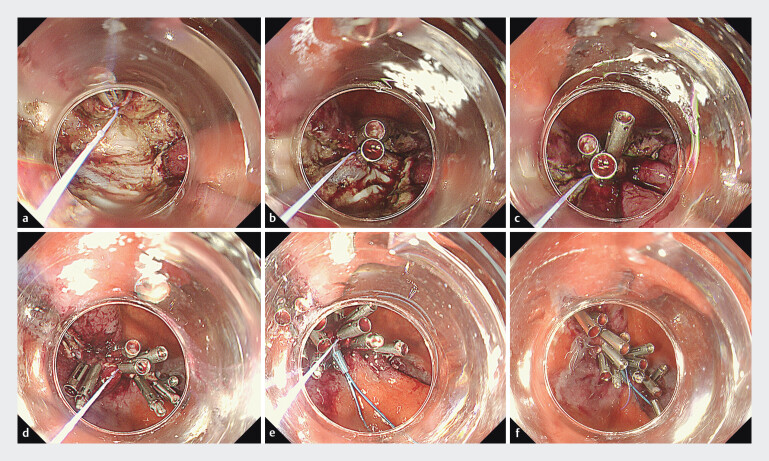
Closure technique. **a** A string clip was placed at the distal edge of the central part of the defect. **b** Toward the opposite edge, several regular clips were placed in the ulcer bed in such a way as to grasp the thread and muscle layers. **c** The central part was closed by pulling the thread. **d** Both sides were closed so that it was sandwiched between the MANTIS clips to strengthen the central part so that it did not open apart. Subsequently, additional regular clips were placed to ensure the complete closure. **e** Finally, a detachable snare was inserted along the string through the instrument channel, and a knot was formed. **f** The string and plastic detachable snare were cut with scissor forceps.

**Fig. 2:**
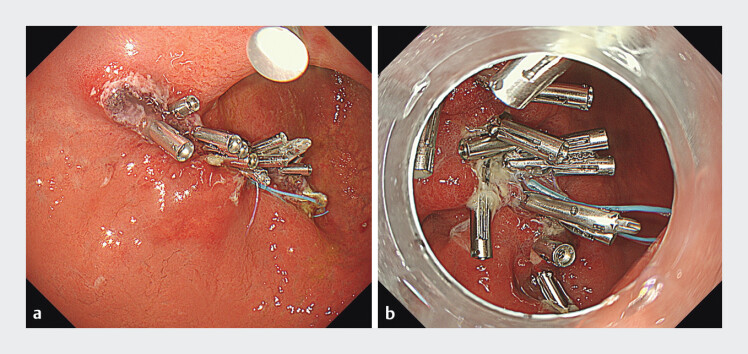
Follow-up endoscopies on postoperative days 1 and 7 confirmed sustained closure. **a** Assessment on postoperative day 1. **b** Assessment on postoperative day 7.

**Fig. 3:**
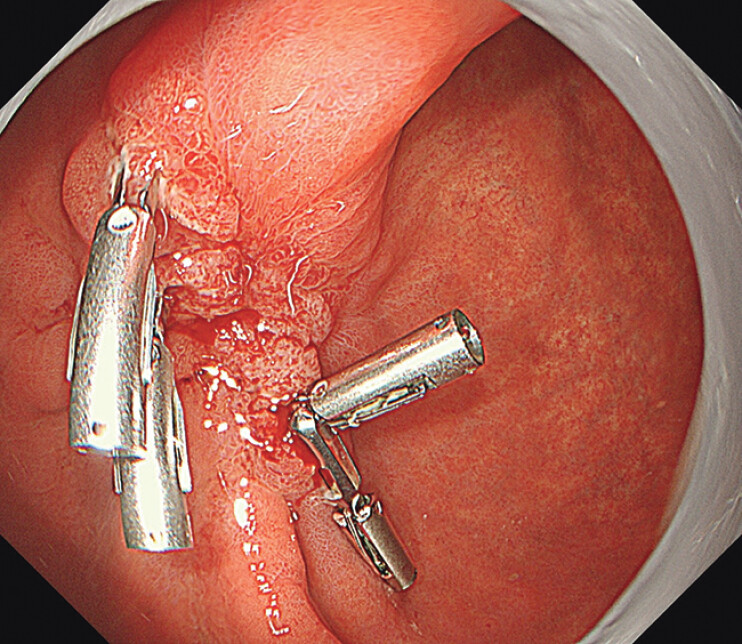
An endoscopy 2 months later confirmed complete scarring and residual presence of four regular clips.

An 82-year-old man underwent standard ESD for a 20-mm early gastric cancer in the anterior wall of the lower body, resulting in an approximately 4-cm defect. After hemostasis of the ulcer bed by coagulation, we decided to close the defect to prevent delayed bleeding. First, a string-clip (clip: SureClip, 16 mm; MicroTech, Nanjing, China) was placed at the distal edge of the central defect ( Fig. 1 **a** ). Several regular clips were placed in the ulcer bed in such a way as to grasp the thread and muscle layers toward the opposite edge ( Fig. 1 **b** ). After pulling the thread and closing the central part ( Fig. 1 **c** ), both sides were closed so that it was sandwiched between two MANTIS clips to strengthen the central part so that it did not open apart ( Fig. 1 **d** ). Additional regular clips were placed as appropriate to achieve complete closure, and finally, a detachable snare (HX-400U-30; Olympus Medical System, Tokyo, Japan) that inserted along the string through an instrument channel was deployed to form a knot ( Fig. 1 **e** ). The string and plastic detachable snare were cut using scissor forceps ( Fig. 1 **f** ). This process of closure was completed within 23 minutes. Follow-up endoscopies on postoperative days 1 and 7 confirmed the sustained effectiveness of the process ( Fig. 2 ). The patient, with no adverse events, was discharged 7 days after the procedure. The subsequent clinical course was good, and an endoscopy 2 months later confirmed complete scarring and the residual presence of four regular clips ( Fig. 3 ).

Strengthening closure of the central part of the defect using a MANTIS-clip, string-clip, and detachable snare (used as an anchor) can potentially prevent subsequent dehiscence and support long-term maintenance of defect closure.
